# Prognostic Value of Hematogones in Patients With Hematopoietic Disorders After Allogeneic Hematopoietic Stem Cell Transplantation: A Systematic Review and Meta-Analysis

**DOI:** 10.7759/cureus.47184

**Published:** 2023-10-17

**Authors:** Hirotaka Mori, Daisuke Koyama, Yuki Sato, Yuki Kataoka, Shunsuke Taito, Takashi Ishio, Takanori Teshima, Isao Yokota

**Affiliations:** 1 Department of Biostatistics, Graduate School of Medicine, Hokkaido University, Sapporo, JPN; 2 Department of Hematology, Hokkaido University Faculty of Medicine, Sapporo, JPN; 3 Department of Systematic Reviewers, Scientific Research Works Peer Support Group, Osaka, JPN; 4 Department of Hematology, Fukushima Medical University, Fukushima, JPN; 5 Department of Healthcare Epidemiology, Kyoto University Graduate School of Medicine, School of Public Health, Kyoto, JPN; 6 Department of Community Medicine, Kyoto University Graduate School of Medicine, Section of Clinical Epidemiology, Kyoto, JPN; 7 Department of Internal Medicine, Kyoto Min-iren Asukai Hospital, Kyoto, JPN; 8 Division of Rehabilitation, Hiroshima University Hospital, Hiroshima, JPN

**Keywords:** grade approach, systematic review, prognostic value, meta-analysis, hematogones

## Abstract

This systematic review and meta-analysis aimed to determine whether hematogones in patients with hematopoietic disorders after allogeneic hematopoietic stem cell transplantation (allo-HSCT) are associated with clinical outcomes.

We searched the MEDLINE, Embase, Cochrane Central Register of Controlled Trials, ClinicalTrials.gov, and World Health Organization International Clinical Trials Registry Platform databases from their inception to March 2023. The primary outcome in the summary of findings was three-year relapse-free survival (RFS), and secondary outcomes in the summary of findings included three-year relapse, non-relapse mortality (NRM), overall survival (OS), acute and chronic graft-versus-host disease (GVHD), and infection. The certainty of evidence was determined using the grading of recommendation assessment, development, and evaluation approaches. A systematic review and meta-analysis of outcome measures were conducted using a random-effects model. This study protocol was registered in the Open Science Framework.

A total of six studies (including 888 patients) were included in the meta-analysis. Hematogones were related to favorable three-year RFS (risk ratio (RR) = 1.84; 95% confidence interval (CI) = 1.01 to 3.34) and favorable NRM (RR = 0.14; 95% CI = 0.04 to 0.51), OS (RR = 1.51; 95% CI = 1.13 to 2.02), and acute GVHD (RR = 0.44; 95% CI = 0.33 to 0.59). The certainty of the evidence was low for RFS, NRM, OS, and acute GVHD. Evidence regarding the association between hematogones, relapse, and infections is uncertain. Hematogones may be a prognostic factor for long-term prognosis and acute adverse events in patients with hematopoietic disorders after allo-HSCT. Further studies are required to address the long-term life-threatening events.

## Introduction and background

Allogeneic hematopoietic stem cell transplantation (allo-HSCT) is an important and potentially curative treatment for many malignant and non-malignant hematopoietic bone marrow disorders, such as aplastic anemia [1]. However, the complications associated with allo-HSCT are critical. For example, acute and chronic graft-versus-host disease (GVHD) manifests as the most prevalent and critical complication [2,3]. Infections pose a substantial risk of fatality during immune reconstruction [4], which may span up to two years [5]. Moreover, the incidence of relapse is as high as 30-40% [6] and remains a significant cause of mortality after allo-HSCT [7]. Accurate prediction and management of these outcomes are essential for successful allo-HSCT [8].

Hematogones, which denote benign B-lymphocyte precursors, appear during bone marrow function recovery after induction therapy, consolidation therapy, or allo-HSCT [9,10]. Hematogones correlate with better overall survival (OS) and relapse-free survival (RFS), and a lower incidence of treatment-related mortality and GVHD in patients after allo-HSCT [11]. Additionally, their presence is a predictor of a lower incidence of infections [12,13]. However, to our knowledge, no systematic review has summarized the evidence across these outcomes or presented the clinical applicability of hematogones.

To investigate the prognostic value of hematogones in the clinical course of allo-HSCT, this systematic review and meta-analysis estimated an average effect across studies and assessed each study’s limitations as well as gaps in the entire published literature. This study also provided information to guide a further study in the context of study design.

## Review

Methodology

Compliance with Reporting Guidelines

Using a pre-specified protocol (Open Science Frame Registry ID: CRD42018105511), we conducted a systematic review of the relevant literature in agreement with the recommendations of the Cochrane Handbook [14] and the Preferred Reporting Items for Systematic Reviews and Meta-Analysis (PRISMA) guidelines [15]. We confirmed that this systematic review was PRISMA-compliant by consulting the PRISMA 2020 checklist [16].

Eligibility Criteria

The target population comprised patients with hematological disorders who underwent allo-HSCT. Hematogones in bone marrow aspiration or biopsy smear are defined as lymphoid precursor cells with diameters varying from 10 to 20 µm, scant cytoplasm, and densely homogeneous nuclear chromatin [9]. The bone marrow tests used to evaluate hematogones include bone marrow aspiration and biopsy. Flow cytometry (FCM) is defined as low-side scatter, intermediate expression of CD45, and bright expression of at least both CD10 and CD19 or terminal deoxynucleotidyl transferase [9]. We adopted the results of positive and negative hematogones reported in each study because there is no clear cutoff value for the percentage of hematogones present in the total bone marrow cells. The number of cells in the bone marrow aspiration smear or events in the FCM analysis was not subjected to inquiry. We set the period for evaluating hematogones to be approximately the time of engraftment, with an allowable range from day 10 to day 100.

The primary outcome was the three-year RFS. The secondary outcomes were the proportion of one-, three-, and five-year relapses; non-relapse mortality (NRM); overall survival (OS); incidence of grade Ⅱ-IV acute GVHD at one year; moderate-to-severe chronic GVHD at three years; total infections at one year; bacterial, viral, and fungal infections at one year; and serum immunoglobulin (Ig)G levels at three months.

We included prospective and retrospective cohort studies that assessed the clinical predictive value of hematogones. No restrictions were applied based on the language of publication, country, observation period, or year of publication. Published articles were included, whereas conference abstracts were excluded.

We excluded patients presenting with the following results from the bone marrow samples: (1) recurrence, (2) residual disease, or (3) incomplete chimerism. The definitions of these were adopted from the respective studies. No exclusion was made based on donor source or disease status at the time of allo-HSCT.

Search Strategy and Selection of Studies

World Health Organization International Clinical Trials Registry Platform and ClinicalTrial.gov. via search portals. The search was performed on March 15, 2023, using a set of suitable search terms, including “allo-HSCT,” “hematogones,” “B-lymphoid precursor cells,” and related terms (Table 1). We also attempted to identify other relevant studies by manually searching the reference lists of included studies. If the candidate study did not contain the necessary information, the authors were contacted to obtain the relevant information. Two reviewers (Hirotaka Mori and Yuki Sato) independently screened the titles and abstracts of each study obtained by the search to determine whether they met the inclusion criteria. The reviewers conducted a full-text review to assess the eligibility of each candidate study. Disagreements were resolved through discussion with a third reviewer (Daisuke Koyama).

**Table 1:** Search strategy.

Database	Search strategies
CENTRAL via Cochrane Library	#1. MeSH descriptor: [Stem Cell Transplantation] explode all trees
	#2. (transplant*):ti,ab,kw
	#3. #1 OR #2
	#4. (Hematogone*):ti,ab,kw
	#5. (Haematogone*):ti,ab,kw
	#6. #4 OR #5
	#7. MeSH descriptor: [Precursor Cells, B-Lymphoid] explode all trees
	#8. (“b cell precursor*”):ti,ab,kw
	#9. (“b lymphoid precursor*”):ti,ab,kw
	#10. (“b lymphocyte precursor*”):ti,ab,kw
	#11. #7 OR #8 OR #9 OR #10
	#12. #6 OR #11
	#13. #3 AND #12
MEDLINE via PubMed	#1. “Stem Cell Transplantation”[MeSH Terms]
	#2. transplant*[Title/Abstract]
	#3. #1 OR #2
	#4. Hematogone*[Title/Abstract]
	#5. Haematogone*[Title/Abstract]
	#6. #4 OR #5
	#7. “Precursor Cells, B-Lymphoid”[MeSH Terms]
	#8. “b cell precursor*”[Title/Abstract]
	#9. “b lymphoid precursor*”[Title/Abstract]
	#10. “b lymphocyte precursor*”[Title/Abstract]
	#11. #7 OR #8 OR #9 OR #10
	#12. #6 OR #11
	#13. #3 AND #12
EMBASE via Dialog	S1. EMB.EXACT.EXPLODE(“stem cell transplantation”)
	S2. TI(transplant*) OR AB(transplant*)
	S3. S1 OR S2
	S4. TI(Hematogone*) OR AB(Hematogone*)
	S5. TI(Haematogone*) OR AB(Haematogone*)
	S6. S4 OR S5
	S7. EMB.EXACT.EXPLODE(“pre B lymphocyte”)
	S8. TI(“b cell precursor*”) OR AB(“b cell precursor*”)
	S9. TI(“b lymphoid precursor*”) OR AB(“b lymphoid precursor*”)
	S10. TI(“b lymphocyte precursor*”) OR AB(“b lymphocyte precursor*”)
	S11. S7 OR S8 OR S9 OR S10
	S12. S6 OR S11
	S13. S3 AND S12
	S14. (S3 AND S12) not (rtype.exact(“Conference Abstract” OR “Conference Paper” OR “Conference Review” OR “Editorial” OR “Chapter”))
WHO ICTRP	in the Title: (b cell precursor* OR hematogone*) AND (stem cell transplantation)
ClinicalTrials.gov	Condition or disease: (“b cell precursors” OR “b cell precursor” OR “hematogones” OR “hematogone”) AND (stem cell transplantation)

Data Extraction and Quality Assessment

Two reviewers (Hirotaka Mori and Yuki Sato) independently extracted study-level data using pre-specified forms. Disagreements regarding data extraction were resolved through discussion. Where necessary, we contacted the authors of the studies for which sufficient information was not available. The risk of bias of each study was assessed independently by two reviewers (Hirotaka Mori and Daisuke Koyama) using the Quality in Prognostic Studies (QUIPS) tool [17]. We assessed the confounding domains using the QUIPS tool. Differences in opinion regarding the assessment of risk of bias were resolved through discussion between the two reviewers.

Data Analysis

For three-year RFS; three-year, one-year, and five-year relapses; NRM; OS; incidence of acute grade II-IV GVHD at one year; moderate-to-severe chronic GVHD at three years; total infections at one year; and bacterial, viral, and fungal infections as dichotomous variables and pooled risk ratios (RR) with 95% confidence interval (CI) were provided. The mean differences with 95% CI were calculated for serum IgG levels as continuous variables.

To calculate the number of events, we multiplied the percentage of events at a prespecified time point using the Kaplan-Meier method [18] or Gray’s method [19] using the effective sample size [20]. The calculations consisted of three steps. First, we reconstructed the order of events and censored the outcomes from published or ordered Kaplan-Meier curves or cumulative incidence curves using DigitizeIt software (version 2.5.9) (http://www.digitizeit.de/) and applied established algorithms [21,22]. Second, we estimated the standard errors of event probabilities from the reconstructed data. Finally, we used the effective sample size obtained from the Greenwood formula to calculate the RR and 95% CI [20]. The effective sample size is a measure of sample size that accounts for the precision obtained from observed right-censored data. Therefore, the effective sample size is smaller than the reported baseline sample size according to the number of censored patients in the original study.

We created a table of summary of findings including the overall grading of evidence certainty for each of the main outcomes, evaluated using the Grading of Recommendations, Assessment, Development, and Evaluation (GRADE) approach [23].

All analyses were conducted using the Stata software version 17 (Stata Corp., College Station, TX, USA).

Results

Study Selection

**Figure 1:**
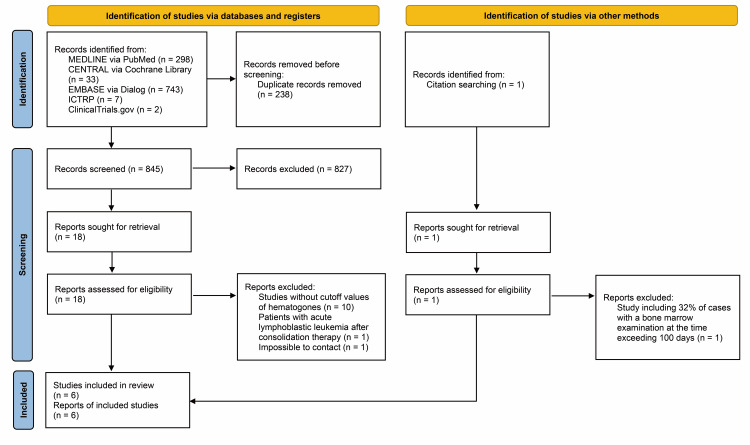
The Preferred Reporting Items for Systematic Reviews and Meta-Analysis 2020 flow diagram. From Page et al. [15].

Figure 1 shows the results of the literature search using the PRISMA 2020 flow diagram [16]. After removing duplicates, we screened 845 records, from which we reviewed 18 full-text documents, excluded 12 studies (Appendices), and, finally, included six articles [11-13,24-26]. Subsequently, we searched the references of the included studies. However, no articles among these fulfilled the inclusion criteria.

Study Characteristics

**Figure 2:**
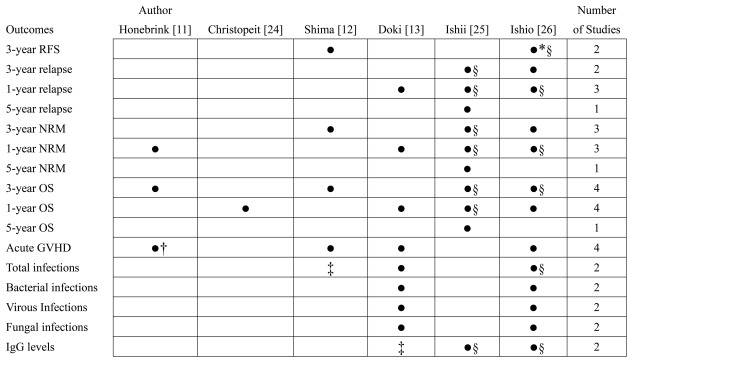
Outcomes of the meta-analysis. ^*^: Subgroup analyses were performed. ^†^: Bone marrow biopsy on day 21 and other outcomes of the Honebrink et al. study on day 100. ^‡^: Unavailable information which our inquiry could not complement. ^§^: Data available upon request to the author. The black circles indicate the outcomes of the study conducted by the authors. RFS = relapse-free survival; NRM = non-relapse mortality; OS = overall survival; GVHD = graft-versus-host disease

Table 2 presents the characteristics of the included studies. The six studies included a total of 888 patients. Two studies defined hematogones based on smear findings [11,25], whereas four defined them based on FMC [12,13,24,26]. The cutoff point of the hematogones varied from 0% to 5%. The percentage of positive hematogones ranged from 26.1% to 75.8%. Table 3 shows the details of the patient characteristics, including disease categories and status, donor sources, preconditioning regimen, GVHD prophylaxis, the follow-up period of survivors, and the test day of hematogones. Figure 2 shows the synthesized results. The extracted outcomes, determined a priori, differed across studies. The one- and three-year OS and acute GVHD outcomes were the most reported outcomes in this meta-analysis of four studies.

**Table 2:** Characteristics of the included studies. Data were presented as numbers (percentages). ND = not described; FCM = flow cytometry; BMT = bone marrow transplantation; CBT = cord blood transplantation

	Honebrink et al. [11]	Christopeit et al. [24]	Shima et al. [12]	Doki et al. [13]	Ishii et al. [25]	Ishio et al. [26]
Publish year	2012	2013	2013	2015	2017	2018
Study types	Prospective	Retrospective	ND	ND	Retrospective	Retrospective
Sample size	Test day 21/100: 86/66	59	108	192	134	309
Hematogones						
Definition test	Smear	FCM	FCM	FCM	Smear	FCM
Cutoff percentage	Test day 21/100: 0/0.9	0.25	5	0.25	1.0	0.1
Positive/Negative	Test day 21: 60/26 (69.8/30.2) Test day 100: 50/16 (75.8/24.2)	24/35 (40.7/59.3)	43/65 (39.8/60.2)	78/114 (40.6/59.4)	35/99 (26.1/73.9)	117/192 (37.9/62.1)
Test days	21 (n = 86) 100 (n = 66)	100		30		
Median			25/32 (BMT/CBT)		41	30
Minimum			15/14 (BMT/CBT)		20	17
Maximum			32/39 (BMT/CBT)		77	71
Note		The number of outcomes on test day 21 was not described			16 patients who relapsed or died within 90 days were excluded	

**Table 3:** Patient characteristics. ^†^: AML or MDS; ^‡^: Tac-based GVHD prophylaxis BMT = bone marrow transplantation; CBT = cord blood transplantation; AML = acute myeloblastic leukemia; MDS = myelodysplastic syndrome; ALL = acute lymphoblastic leukemia; CML = chronic myeloblastic leukemia; ML = malignant lymphoma; CR = complete remission; ND = not described; NA = not applicable; PBSCT = peripheral blood stem cell transplantation; MAC = myeloablative conditioning; RIC = reduced-intensity conditioning; Tac = tacrolimus; sMTX = short-term methotrexate; MMF = mycophenolate mofetil; PTCY = post-transplant cyclophosphamide; FCM = flow cytometry

	Honebrink et al. [11]	Christopeit et al. [24]	Shima et al. [12]	Doki et al. [13]	Ishii et al. [25]	Ishio et al. [26]
Diseases categories						
AML	88/66 (Test day 21/100)	63	60†	89	70	124
MDS		13		33	16	41
ALL			18	45	33	71
CML				7	6	13
ML			30	10	7	60
Others				8	2	
Disease status						
CR	86/66 (test day 21/100)	36	41	109		215
Non-CR/NA	2/0	40	67	76		94
Donor sources						
BMT		1	49	192		172
PBSCT		74	NA			88
CBT	88	1	49		134	48
Preconditioning regimens						
MAC	88	25	66	154	127*	158
RIC		51	42	38	7*	151
GVHD prophylaxis	ND					
Tac and sMTX			57	136		192
CsA and sMTX		13	36	56	128	77
Tac and MMF						11
CsA and MMF		59	15		6	2
PTCY						27
Others/unknown		4				
Follow-up period for survivors	ND	ND	ND			
Median (days)				605	78 months	923
Minimum range				31	3 moths	71
Maximum range				3252	153 months	4188

Risk of Bias in the Included Studies

**Figure 3:**
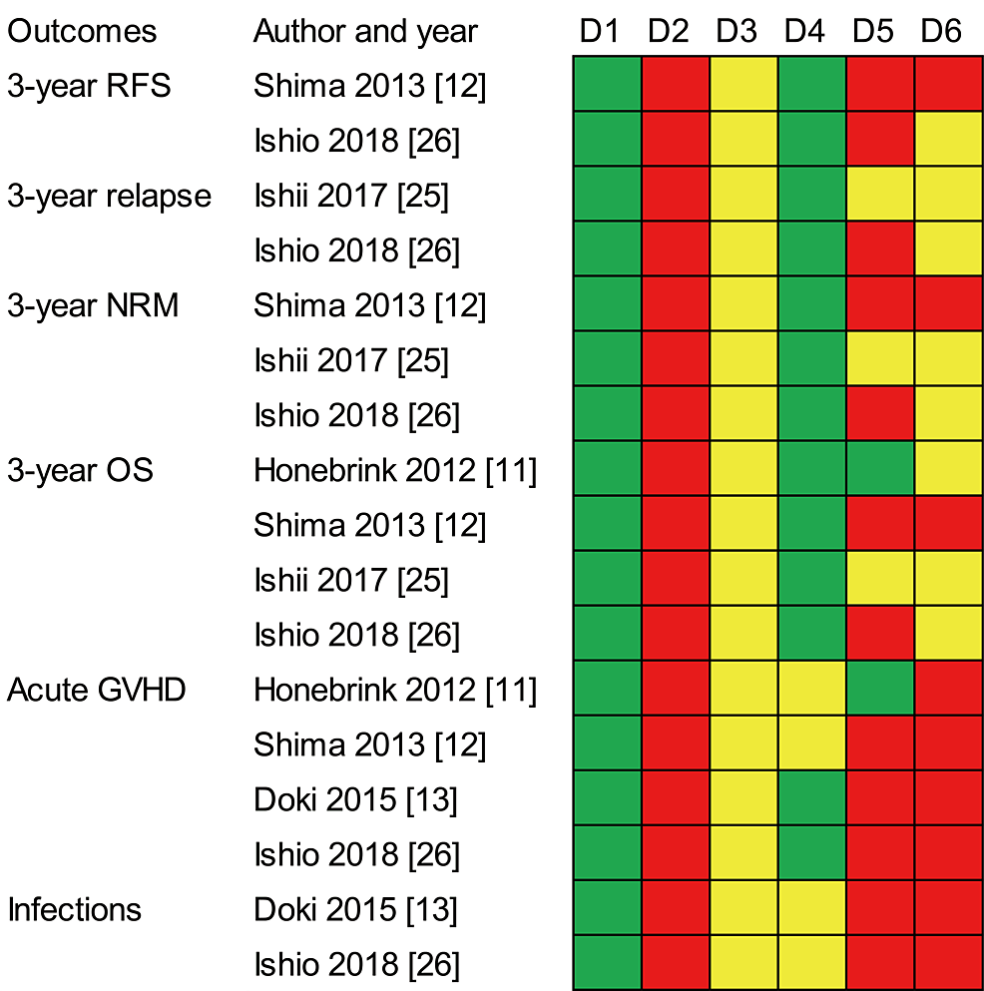
Risk of bias for the summary of outcome findings. D1: study participation; D2: study attrition; D3: prognostic factor measurement; D4: outcome measurement; D5: study confounding; D6: statistical analysis and reporting. The red, yellow, and green blocks indicate high, moderate, and low risk, respectively. RFS = relapse-free survival; NRM = non-relapse mortality; OS = overall survival; GVHD = graft-versus-host disease

The QUIPS tool was used to assess the risk of bias of the included studies [17]. Figure 3 shows the risk of bias in the summary of the outcome findings. The risk of bias was assessed in five studies with a summary of the outcomes [11-13,25,26]. All five studies demonstrated a low risk of bias for study participation; however, all had a high risk of bias for study attrition and a moderate risk of bias for prognostic factor measurements. All five studies had a low risk of bias for outcome measurement of three-year RFS, relapse, NRM, and OS; however, a moderate risk of bias for outcome measurement was found in half of the four studies on acute GVHD [11,12] and all two studies on total infections [13,26]. There was no low risk of bias for study confounders, statistical analysis, or reporting.

Certainty of Evidence

**Figure 4:**
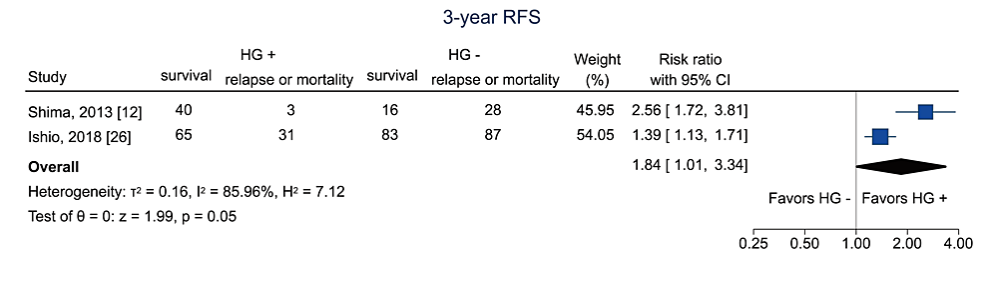
Forest plot of three-year RFS. RFS = relapse-free survival; HG = hematogones; + = positive; - = negative

**Figure 5:**
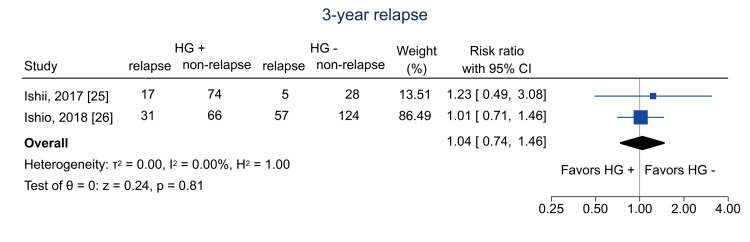
Forest plot of three-year relapse. HG = hematogones; + = positive; - = negative

**Figure 6:**
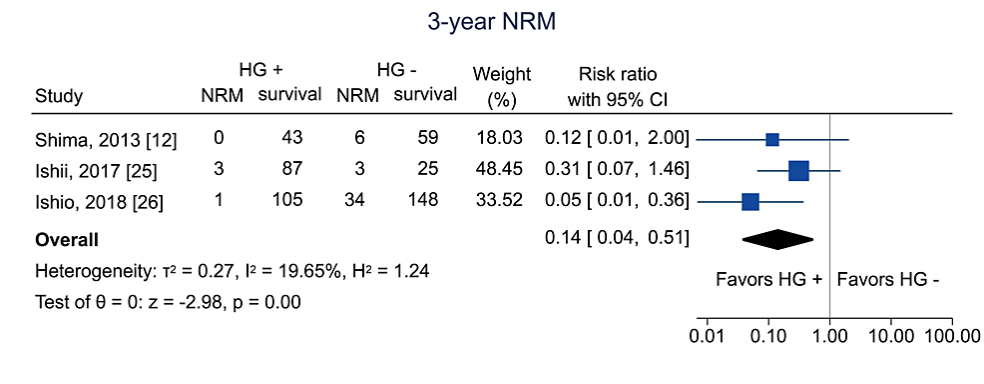
Forest plot of three-year NRM. NRM = non-relapse mortality; HG = hematogones; + = positive; - = negative

**Figure 7:**
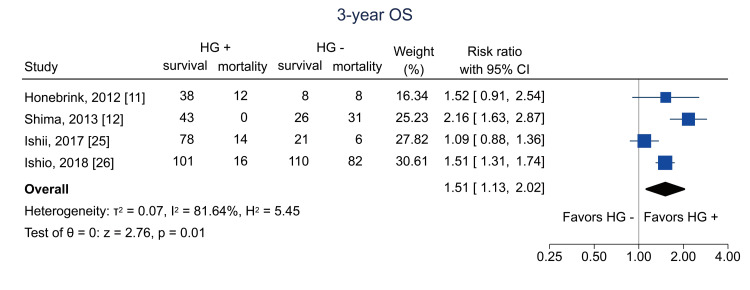
Forest plot of three-year OS. OS = overall survival; HG = hematogones; + = positive; - = negative

**Figure 8:**
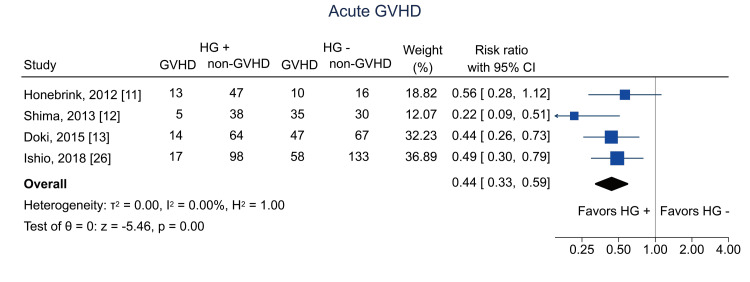
Forest plot of acute GVHD. GVHD = graft-versus-host disease; HG = hematogones; + = positive; - = negative

**Figure 9:**
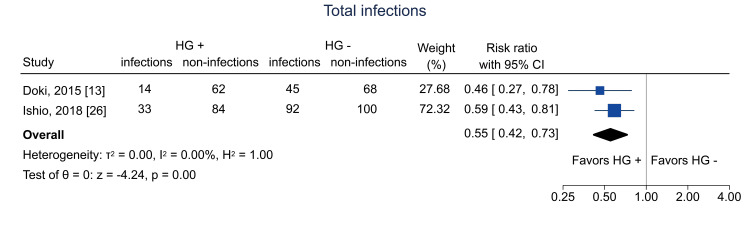
Forest plot of total infections. HG = hematogones; + = positive; - = negative

Table 4 shows the summary of findings. The primary outcome of three-year RFS was reported in two studies [12,26]. Hematogones were significantly related to three-year RFS (RR = 1.84; 95% CI = 1.01 to 3.34; I^2^ = 86.0%; n = 353) (Figure 4); the certainty of the evidence was low. Regarding secondary outcomes, three-year relapse was analyzed in two studies [25,26]; the pooled RR was 1.04 (95% CI = 0.74 to 1.46; I^2^ = 0%; n = 402) (Figure 5), and the certainty of the evidence was very low. Three-year NRM was analyzed in three studies [12,25,26]; the pooled RR was 0.14 (95% CI = 0.04 to 0.51; I^2^ = 19.7%; n = 514) (Figure 6), and the certainty of the evidence was low. Three-year OS was analyzed in four studies [11,12,25,26]; the pooled RR was 1.51 (95% CI = 1.13 to 2.02; I^2^ = 81.6%; n = 594) (Figure 7), and the certainty of the evidence was low. Acute GVHD was analyzed in four studies [11-13,26]; the pooled RR was 0.44 (95% CI = 0.33 to 0.59; I^2^ = 0%; n = 692) (Figure 8), and the certainty of the evidence was low. No study reported the outcome of chronic GVHD, as defined in the protocol. Infections were analyzed in two studies [13,26]; the pooled RR was 0.55 (95% CI = 0.42 to 0.73; I^2^ = 0%; n = 498) (Figure 9), and the certainty of the evidence was very low.

**Table 4:** Summary of findings from six studies focused on hematogones in patients after allo-HSCT. ^*^: The corresponding risk (and its 95% CI) is based on the assumed risk in the comparison group and the relative effect (and its 95% CI) estimated for the intervention group. Assumed risk was median of control risks. GRADE Working Group grades of evidence: High certainty: We are very confident that the true effect lies close to that of the estimate of the effect. Moderate certainty: We are moderately confident in the effect estimate; the true effect is likely to be close to the estimate of the effect, but there is a possibility that it is substantially different. Low certainty: Our confidence in the effect estimate is limited; the true effect may be substantially different from the estimate of the effect. Very low certainty: We have very little confidence in the effect estimate; the true effect is likely to be substantially different from the estimate of effect ^a^: We did not consider the domain of study confounding and statistical analysis and reporting in the risk of bias for judging the certainty of the evidence. ^b^: Downgraded one point because of moderate or high risk of bias associated with attrition, prognostic factor measurement, and outcome measurement. ^c^: Downgraded one point as imprecise (few studies). ^d^: Downgraded two points as imprecise (confidence interval). ^e^: Downgraded two points because of moderate or high risk of bias associated with attrition, prognostic factor measurement, and outcome measurement. allo-HSCT = allogeneic hematopoietic stem cell transplantation; CI = confidence interval; RR = risk ratio; NA = not applicable; RFS = relapse-free survival; NRM = non-relapse mortality; OS = overall survival; GVHD = graft versus host disease; NA = not applicable

Outcome	Illustrative comparative risks^*^ (95% CI)		Relative effect (95% CI)	Number of participants (studies)	Certainty of the evidence^ a^ (GRADE)
	Assumed risk	Corresponding risk			
	Comparison	Exposure			
Three-year RFS	Study population		RR 1.84 (1.01 to 3.34)	353 (2 studies)	⊕⊕⊝⊝ Low^ b, c^
	463 per 1,000	852 per 1,000 (468 to 1,000)			
Three-year relapse	Study population		RR 1.04 (0.74 to 1.46)	402 (2 studies)	⊕⊝⊝⊝ Very low^ b, d^
	290 per 1,000	302 per 1,000 (215 to 423)			
Three-year NRM	Study population		RR 0.14 (0.04 to 0.51)	514 (3 studies)	⊕⊕⊝⊝ Low^ b, c ^
	107 per 1,000	15 per 1,000 (4 to 55)			
Three-year OS	Study population		RR 1.51 (1.13 to 2.02)	594 (4 studies)	⊕⊕⊝⊝ Low ^b, c^
	537 per 1,000	811 per 1,000 (607 to 1,000)			
Acute GVHD	Study population		RR 0.44 (0.33 to 0.59)	692 (4 studies)	⊕⊕⊝⊝ Low ^b, c^
	398 per 1,000	175 per 1,000 (131 to 235)			
Chronic GVHD	Study population		NA	NA	NA
	NA	NA			
Infections	Study population		RR 0.55 (0.42 to 0.73)	498 (2 studies)	⊕⊝⊝⊝ Very low ^c, e ^
	439 per 1,000	241 (184 to 321)			

Subgroup Analysis

The prespecified subgroup analysis for the primary outcome of three-year RFS revealed a similar direction of effect to the overall group [26]. We observed no notable differences among the bone marrow (RR = 1.28; 95% CI = 1.00 to 1.65), peripheral blood stem cell (RR = 1.70; 95% CI = 1.14 to 2.52), and cord blood transplantation subgroups (RR = 1.38; 95% CI = 0.80 to 2.40). We also observed no notable differences among disease categories of acute myeloid leukemia or myelodysplastic syndromes (RR = 1.15; 95% CI = 0.77 to 1.74), acute lymphoblastic leukemia (RR = 1.41; 95% CI = 0.96 to 2.07), and lymphoma (RR = 1.43; 95% CI = 0.95 to 2.14).

Additional Outcomes

**Figure 10:**
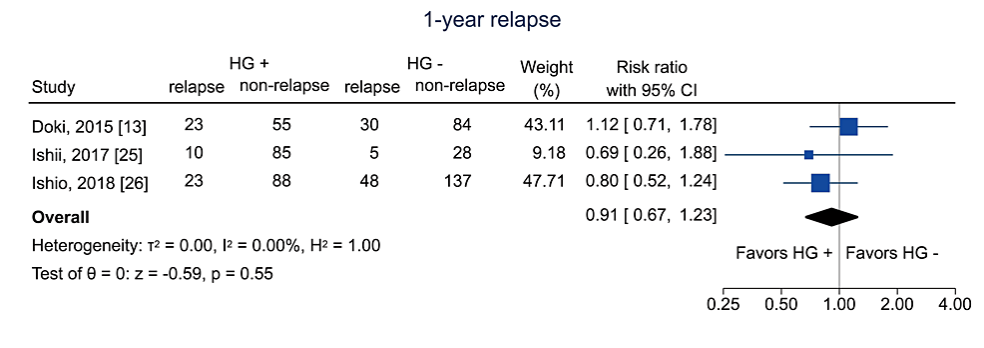
Forest plot of one-year relapse. HG = hematogones; + = positive; - = negative

**Figure 11:**
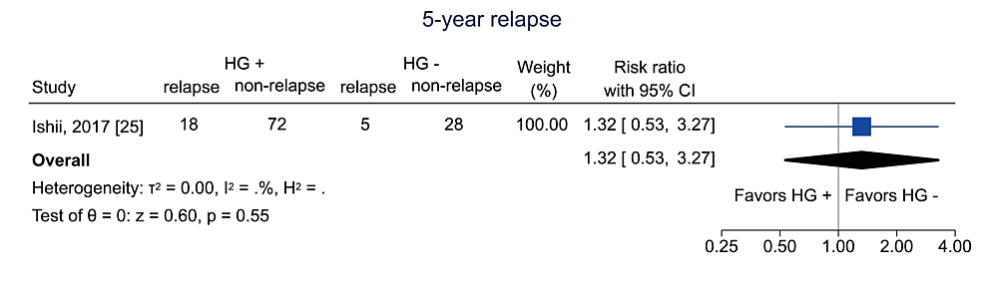
Forest plot of five-year relapse. HG = hematogones; + = positive; - = negative

Data on one-year relapse were available from three studies [13,25,26]; the pooled RR was 0.91 (95% CI = 0.67 to 1.23; I^2^ = 0%; n = 616) (Figure 10). Data on five-year relapse were available from one study [25]; the RR was 1.32 (95% CI = 0.53 to 3.27; I^2^ = NA; n = 123) (Figure 11).

**Figure 12:**
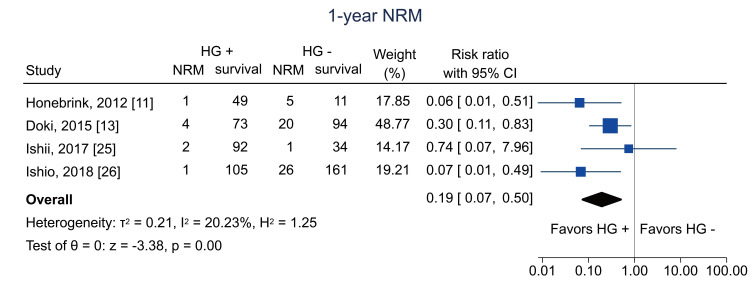
Forest plot of one-year NRM. HG = hematogones; + = positive; - = negative; NRM = non-relapse mortality

**Figure 13:**
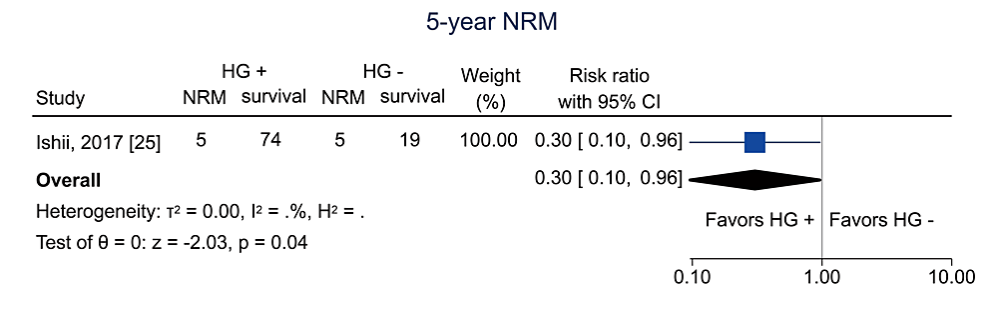
Forest plot of five-year NRM. HG = hematogones; + = positive; - = negative; NRM = non-relapse mortality

Data on one-year NRM were available from four studies [11,13,25,26]; the pooled RR was 0.19 (95% CI = 0.07 to 0.50; I^2^ = 20.2%; n = 679) (Figure 12). Data on five-year NRM were obtained from one study [25]; the RR was 0.30 (95% CI = 0.10 to 0.96; I^2^ = NA; n = 103) (Figure 13).

**Figure 14:**
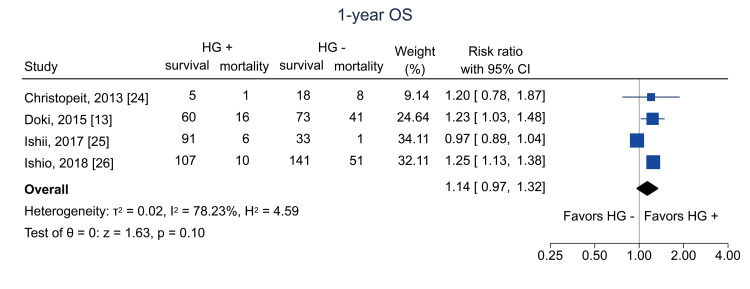
Forest plot of one-year OS. HG = hematogones; + = positive; - = negative; OS = overall survival

**Figure 15:**
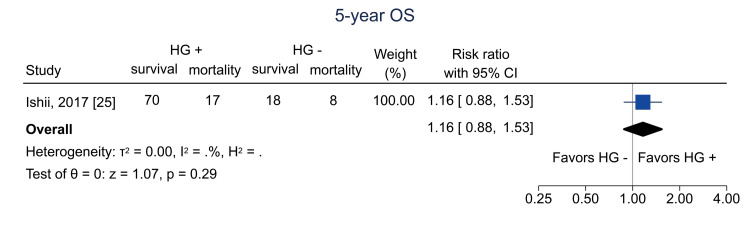
Forest plot of five-year OS. HG = hematogones; + = positive; - = negative; OS = overall survival

Data on one-year OS were available from four studies [13,24-26]; the pooled RR was 1.14 (95% CI = 0.97 to 1.32; I^2^ = 78.2%; n = 662) (Figure 14). Data on five-year OS were available from one study [25]; the RR was 1.16 (95% CI = 0.88 to 1.53; I^2^ = NA; n = 113) (Figure 15).

**Figure 16:**
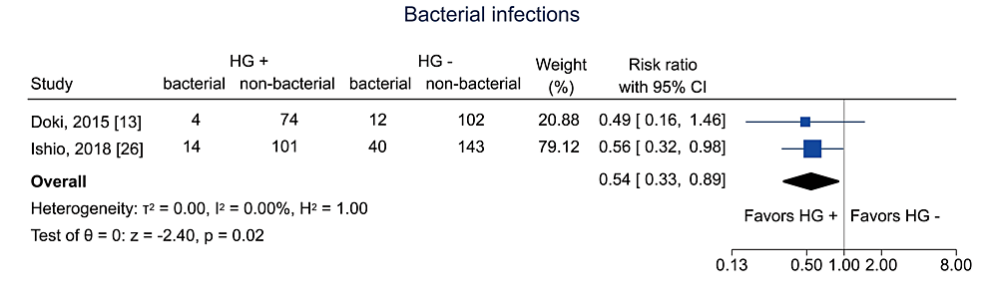
Forest plot of bacterial infections. HG = hematogones; + = positive; - = negative

**Figure 17:**
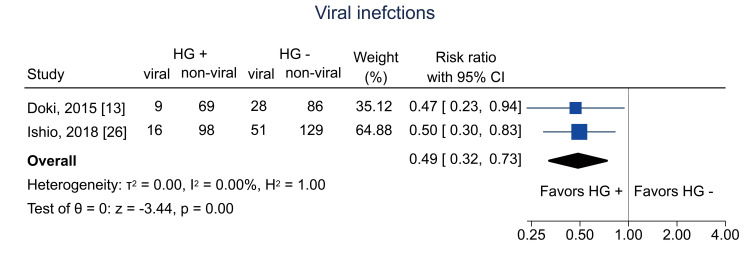
Forest plot of viral infections. HG = hematogones; + = positive; - = negative

**Figure 18:**
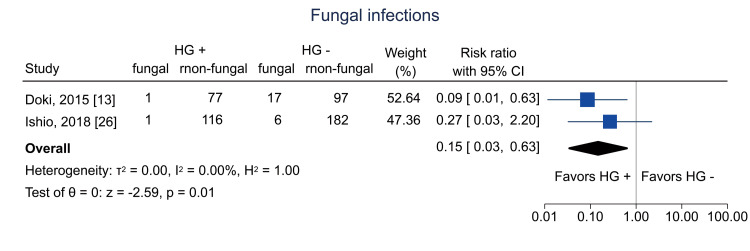
Forest plot of fungal infections. HG = hematogones; + = positive; - = negative

**Figure 19:**
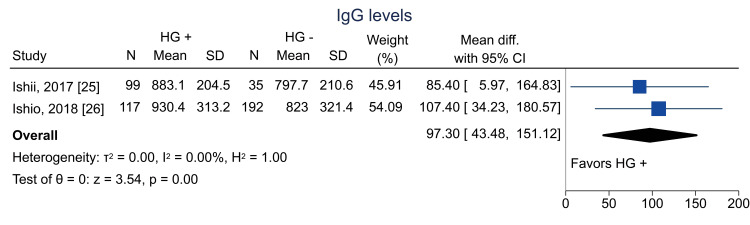
Forest plot of IgG levels. IgG = immunoglobulin G; HG = hematogones; + = positive; - = negative

Data on bacterial infections at one year were available from two studies [13,26]; the pooled RR was 0.54 (95% CI = 0.33 to 0.89; I^2^ = 0%; n = 490) (Figure 16). Data on viral infections at one year were available from two studies [13,26]; the pooled RR was 0.49 (95% CI = 0.32 to 0.73; I^2^ = 0%; n = 486) (Figure 17). Data on fungal infections at one year were available from two studies [13,26]; the pooled RR was 0.15 (95% CI = 0.03 to 0.63; I^2^ = 0%; n = 497) (Figure 18). Data on IgG levels at three months were available in two studies [25,26]; the mean difference was 97.3 mg/dL (95% CI = 43.5 to 151.1; I^2^ = 0%; n = 443) (Figure 19).

Differences Between the Protocol and the Review

As the largest number of integrated studies was four, we could not assess publication bias using funnel plots and Egger tests. Owing to the small sample size, we could not conduct planned sensitivity analyses for the primary outcomes. (i) exclusion of studies with a high risk in QUIPS domains, (ii) exclusion of the prognostic values of hematogones evaluated from 100 median days after allo-HSCT, and (iii) when the meta-analytic result of the primary outcome showed significant heterogeneity. We could not perform a meta-regression of the cutoff levels for positive or negative hematogones because of the small number of included studies. We also searched for case-control studies but found none that met the eligibility criteria.

Discussion

This systematic review included six studies evaluating the prognostic value of hematogones in 888 patients with hematopoietic disorders after allo-HSCT. The presence of hematogones was related to lower cumulative incidence estimates of three-year NRM, and, as a result, demonstrated an improvement in three-year RFS. Subgroup analysis by donor source and disease category consistently demonstrated a favorable three-year RFS. Acute GVHD and infections were the major causes of NRM in the allo-HSCT setting [27,28], and less acute GVHD and infections translated into lower NRM. Additionally, our results on RFS, NRM, acute GVHD, and infections suggest a favorable OS in patients with positive hematogones [29]. The benefits of less acute GVHD and fewer infections in patients with hematogones may outweigh the uncertain effects of relapse.

Our results imply that hematogones are a prognostic factor for survival and acute GVHD in patients with hematopoietic disorders after allo-HSCT. Currently, the useful prognostic factors for patients in complete remission after allo-HSCT are limited. A cohort study showed that the prognostic values of neutrophil and platelet engraftment times for the three-year OS rate were almost the same in patients with engraftment before day 28 and those with engraftment between days 28 and 42 [30]. A meta-analysis showed that engraftment syndrome (ES) was a negative prognostic factor, and the incidence of ES was associated with a higher incidence of acute and chronic GVHD, NRM, and decreased OS compared with the absence of ES [31]. Our meta-analysis confirmed that hematogones are a prognostic factor for favorable survival and acute adverse outcomes in patients in complete remission. These positive prognostic values indicate the potential therapeutic strategies. The prevalence of hematogones further supports the clinical decision of physicians to follow a consensus on the use of low-dose steroids for the management of grade 2 or lower acute GVHD with isolated cutaneous and upper gastrointestinal symptoms [32]. In cases of first-line systemic therapy for acute GVHD [33], slow tapering rates of steroid doses to avoid exacerbation or recurrence of acute GVHD might be one of the acceptable ways considering the low risk of primary disease recurrence and infection in patients with hematogones.

The evidence of hematogones is uncertain regarding long-term adverse events, such as relapse and infection, and there is no information about chronic GVHD. In particular, results regarding relapse, in which the confidence interval crosses the line of null effects, should be interpreted with caution, although this does not mean that hematogones have no prognostic value [34]. The significant effect sizes of bacterial, viral, and fungal infections and serum IgG levels did not reinforce confidence in the results regarding total infections. Severe chronic GVHD can be fatal following allo-HSCT [2]. However, no study has reported the grade of chronic GVHD according to the National Institutes of Health criteria [35]. Further studies are required to evaluate the grade of chronic GVHD based on these criteria.

This review has several strengths. The results of this review are based on the best available evidence after a comprehensive search. Additionally, we used a systematic methodology that followed a written a priori protocol developed according to the PRISMA statement [16]. We used the GRADE approach [23] to assess the certainty of evidence. This method strengthens the recommendations for the use of hematogones as a prognostic factor in the current clinical practice of allo-HSCT.

There are some potential limitations to the risk of bias and study methods. First, attrition bias was judged to be high in all studies. The included studies did not report the number of participants lost to follow-up or the reasons, which could have systematically biased the association between the prognostic factors and prognosis [36]. Additionally, the number of events was estimated using the Kaplan-Meier and Gray’s method [18,19] because no other information was available. This method tends to overestimate the number of events to the extent that patients are lost to follow-up or censored [37]. However, to address the issue of overestimated event proportions, we widened the 95% CI using an effective sample size to account for censoring [20]. Second, the data extracted for the meta-analysis were reported in crude form and were not adjusted for the various confounders listed in Table 3, such as disease status at allo-HSCT, human leukocyte antigen match, preconditioning regimen, and GVHD prophylaxis [13,25,26]. The adjusted effect size may have reduced the borderline significance of our meta-analysis [38]. Third, the variation in the cutoff levels of hematogones between studies may have influenced the results. We planned to perform a meta-regression to address this heterogeneity but were unable to do so because of the limited number of studies that included a subgroup analysis. Further research is needed to address the high attrition bias and loss to follow-up, unadjusted reports of confounding factors, and optimal cutoff levels of hematogones.

## Conclusions

Hematologists may use hematogones as prognostic factors for long-term prognosis and acute unfavorable events in patients with hematopoietic disorders following allo-HSCT. There is still room for improvement in methods of primary research to lower the risk of bias. Further cohort studies are warranted to address the methodological issues and the uncertainty regarding long-term life-threatening events.

## Appendices

**Table 5:** Characteristics of excluded studies.

Reason for exclusion	Study
Studies without cutoff values of hematogones	Storek et al. Am J Hematol. 1996; 52: 82-9 Storek et al. Blood. 2001; 98: 489-91. Sánchez-García et al. Haematologica. 2006; 91: 340-7. Kahng et al. Korean J Lab Med. 2007; 27: 406-13 Fedoriw et al. Biol Blood Marrow Transplant. 2012;18: 968-73. Mensen et al. Blood. 2014; 124: 963-72 Sarvaria et al. Blood. 2016; 128: 1346-61. Bohmann et al. Ann Hematol. 2017; 96: 299-310. Skert et al. PLoS One. 2017; 12: e0175337. Schultz KR, Blood. 2020; 135: 1287-98
Impossible to contact	Hagino et al. Tokyo Jikeikai Med J. 2006; 121: 17-26
Patients with acute lymphoblastic leukemia after consolidation therapy	Wang et al. Int J Lab Hematol. 2016; 38: 246-55
Study including 32% of cases with a bone marrow examination at a time exceeding 100 days	Montesinos et al. Biol Blood Marrow Transplant. 2012; 18: 388-95

## References

[1] Appelbaum FR (2007). Hematopoietic-cell transplantation at 50. N Engl J Med.

[2] Moon JH, Sohn SK, Lambie A (2014). Validation of National Institutes of Health global scoring system for chronic graft-versus-host disease (GVHD) according to overall and GVHD-specific survival. Biol Blood Marrow Transplant.

[3] Zeiser R, Blazar BR (2017). Acute graft-versus-host disease - biologic process, prevention, and therapy. N Engl J Med.

[4] Sahin U, Toprak SK, Atilla PA, Atilla E, Demirer T (2016). An overview of infectious complications after allogeneic hematopoietic stem cell transplantation. J Infect Chemother.

[5] Ogonek J, Kralj Juric M, Ghimire S, Varanasi PR, Holler E, Greinix H, Weissinger E (2016). Immune reconstitution after allogeneic hematopoietic stem cell transplantation. Front Immunol.

[6] Devillier R, Crocchiolo R, Etienne A (2013). Outcome of relapse after allogeneic stem cell transplant in patients with acute myeloid leukemia. Leuk Lymphoma.

[7] D'Souza A, Fretham C, Lee SJ (2020). Current use of and trends in hematopoietic cell transplantation in the United States. Biol Blood Marrow Transplant.

[8] Holtan SG, DeFor TE, Lazaryan A (2015). Composite end point of graft-versus-host disease-free, relapse-free survival after allogeneic hematopoietic cell transplantation. Blood.

[9] Sevilla DW, Colovai AI, Emmons FN, Bhagat G, Alobeid B (2010). Hematogones: a review and update. Leuk Lymphoma.

[10] Chantepie SP, Salaün V, Parienti JJ (2011). Hematogones: a new prognostic factor for acute myeloblastic leukemia. Blood.

[11] Honebrink T, Dayton V, Burke MJ (2012). Impact of bone marrow hematogones on umbilical cord blood transplantation outcomes in patients with acute myeloid leukemia. Biol Blood Marrow Transplant.

[12] Shima T, Miyamoto T, Kikushige Y (2013). Quantitation of hematogones at the time of engraftment is a useful prognostic indicator in allogeneic hematopoietic stem cell transplantation. Blood.

[13] Doki N, Haraguchi K, Hagino T (2015). Clinical impact of hematogones on outcomes of allogeneic hematopoietic stem cell transplantation. Ann Hematol.

[14] Higgins JP, Thomas J, Chandler J, Cumpston M, Li T, Page MJ, Welch VA (2019). Cochrane Handbook for Systematic Reviews of Interventions. John Wiley & Sons, Chichester.

[15] Page MJ, Moher D, Bossuyt PM (2021). PRISMA 2020 explanation and elaboration: updated guidance and exemplars for reporting systematic reviews. BMJ.

[16] Page MJ, McKenzie JE, Bossuyt PM (2021). The PRISMA 2020 statement: an updated guideline for reporting systematic reviews. BMJ.

[17] Hayden JA, van der Windt DA, Cartwright JL, Côté P, Bombardier C (2013). Assessing bias in studies of prognostic factors. Ann Intern Med.

[18] Kaplan EL, Meier P (1958). Nonparametric estimation from incomplete observations. J Am Stat Assoc.

[19] Gray RJ (1988). A class of K-sample tests for comparing the cumulative incidence of a competing risk. Ann Stat.

[20] Cutler SJ, Ederer F (1958). Maximum utilization of the life table method in analyzing survival. J Chronic Dis.

[21] Guyot P, Ades AE, Ouwens MJ, Welton NJ (2012). Enhanced secondary analysis of survival data: reconstructing the data from published Kaplan-Meier survival curves. BMC Med Res Methodol.

[22] Liu N, Zhou Y, Lee JJ (2021). IPDfromKM: reconstruct individual patient data from published Kaplan-Meier survival curves. BMC Med Res Methodol.

[23] Guyatt G, Oxman AD, Akl EA (2011). GRADE guidelines: 1. Introduction-GRADE evidence profiles and summary of findings tables. J Clin Epidemiol.

[24] Christopeit M, Heiland A, Binder M (2013). Impact of physiological BM CD10+CD19+ B-cell precursors (haematogones) in the post-transplant period in patients with AML. Bone Marrow Transplant.

[25] Ishii H, Konuma T, Kato S, Oiwa-Monna M, Tojo A, Takahashi S (2017). Impact of hematogones on the long-term outcomes of single-unit cord blood transplantation for adult patients. Leuk Lymphoma.

[26] Ishio T, Sugita J, Tateno T (2018). Hematogones predict better outcome in allogeneic hematopoietic stem cell transplantation irrespective of graft sources. Biol Blood Marrow Transplant.

[27] Kurosawa S, Yakushijin K, Yamaguchi T (2013). Recent decrease in non-relapse mortality due to GVHD and infection after allogeneic hematopoietic cell transplantation in non-remission acute leukemia. Bone Marrow Transplant.

[28] Ferrara JL, Levine JE, Reddy P, Holler E (2009). Graft-versus-host disease. Lancet.

[29] McDonald GB, Sandmaier BM, Mielcarek M (2020). Survival, nonrelapse mortality, and relapse-related mortality after allogeneic hematopoietic cell transplantation: comparing 2003-2007 versus 2013-2017 cohorts. Ann Intern Med.

[30] Davies SM, Kollman C, Anasetti C (2000). Engraftment and survival after unrelated-donor bone marrow transplantation: a report from the national marrow donor program. Blood.

[31] ElGohary G, Toor AA, Gergis U (2023). Engraftment syndrome after allogeneic stem cell transplantation: a systematic review and meta-analysis. Bone Marrow Transplant.

[32] Penack O, Marchetti M, Ruutu T (2020). Prophylaxis and management of graft versus host disease after stem-cell transplantation for haematological malignancies: updated consensus recommendations of the European Society for Blood and Marrow Transplantation. Lancet Haematol.

[33] Martin PJ, Rizzo JD, Wingard JR (2012). First- and second-line systemic treatment of acute graft-versus-host disease: recommendations of the American Society of Blood and Marrow Transplantation. Biol Blood Marrow Transplant.

[34] Alderson P (2004). Absence of evidence is not evidence of absence. BMJ.

[35] Filipovich AH, Weisdorf D, Pavletic S (2005). National Institutes of Health consensus development project on criteria for clinical trials in chronic graft-versus-host disease: I. Diagnosis and staging working group report. Biol Blood Marrow Transplant.

[36] Kristman V, Manno M, Côté P (2004). Loss to follow-up in cohort studies: how much is too much?. Eur J Epidemiol.

[37] Duchateau L, Collette L, Sylvester R, Pignon JP (2000). Estimating number of events from the Kaplan-Meier curve for incorporation in a literature-based meta-analysis: what you don't see you can't get!. Biometrics.

[38] Liu T, Nie X, Wu Z (2017). Can statistic adjustment of OR minimize the potential confounding bias for meta-analysis of case-control study? A secondary data analysis. BMC Med Res Methodol.

